# Dietary Niacin Intake Predicts the Decrease of Liver Fat Content During a Lifestyle Intervention

**DOI:** 10.1038/s41598-018-38002-7

**Published:** 2019-02-04

**Authors:** Katarzyna Linder, Caroline Willmann, Konstantinos Kantartzis, Jürgen Machann, Fritz Schick, Marjo Graf, Sabine Kümmerle, Hans-Ulrich Häring, Andreas Fritsche, Norbert Stefan, Róbert Wagner

**Affiliations:** 1 Department of Internal Medicine IV, University Hospital of Tübingen, Tübingen, Germany; 2 Institute of Diabetes Research and Metabolic Diseases (IDM) of the Helmholtz Zentrum München at the University of Tübingen, Tübingen, Germany; 3 Deutsches Zentrum für Diabetesforschung, Neuherberg, Germany; 4 Section on Experimental Radiology, University Hospital of Tübingen, Tübingen, Germany

## Abstract

Niacin inhibits fatty acid flux from adipose tissue to liver, reduces hepatic triglyceride synthesis and increases hepatic lipid oxidation. Thus, niacin may have a role in the regulation of liver fat content in humans. We tested if dietary intake of niacin predicts change of liver fat content during a lifestyle intervention. To this end, we estimated the composition of diet from diaries of 202 healthy subjects at risk of type 2 diabetes undergoing lifestyle intervention comprising physical activity and diet counselling. Total-, subcutaneous- and visceral adipose tissue mass were measured by magnetic resonance (MR) tomography and liver fat content by ^1^H-MR spectroscopy at baseline and after 9 months of follow-up. Among fat compartments, liver fat content showed the largest decrease (−32%, p < 0.0001). High baseline niacin intake predicted a larger decrease of liver fat (p = 0.004). Subjects in the highest quartile of niacin intake at baseline also had the largest decrease of liver fat (1^st^:−10%; 2^nd^:−27%; 3^rd^:−35%; 4^th^:−37%). Among 58 subjects with nonalcoholic fatty liver disease (NAFLD) at baseline, NAFLD resolved in 23 subjects during the lifestyle intervention. For one standard deviation increase in niacin intake, the odds ratio for resolution of NAFLD was 1.77 (95% CI, 1.00–3.43). High dietary niacin intake may have a favorable effect on the reduction of liver fat during lifestyle intervention.

## Introduction

Nonalcoholic fatty liver disease (NAFLD) is the most common chronic liver disease in developed countries, affecting more than one-quarter of adults, and its prevalence is continuing to increase in parallel with the epidemic of obesity^1^. NAFLD is not only a potential precursor for the development of advanced liver diseases but also represents a strong risk factor for the development of insulin resistance, type 2 diabetes and cardiovascular events^2–6^. Thus, the prevention and treatment of fatty liver has high priority, not only on a clinical, but also on a population level.

While pharmacological treatment of NAFLD is under intensive investigation and some studies showed promising results^7^, lifestyle intervention with increase in physical activity and modification of the diet so far represents the most effective and safe approach to prevent and treat NAFLD^8,9^. A plausible mechanism responsible for the beneficial effect of lifestyle intervention on the change in liver fat content is the loss of fat mass, particularly visceral fat, resulting in a reduced portal free fatty acid supply to the liver and favorable changes in secretion of adipocytokines. However, because the decrease in total body- or visceral fat mass is not necessarily accompanied by a decrease in liver fat content, other mechanisms may also be involved in this process^9^. In support, we found that a higher cardiorespiratory fitness at baseline predicted a larger decrease in liver fat content during a lifestyle intervention, independently of total adiposity, body fat distribution and exercise intensity^8^.

Because considerable weight loss and maintenance of reduced body weight in the long term is difficult to achieve, and a high cardiorespiratory fitness is largely genetically determined^10^, it seems highly important to investigate the potential impact of nutrition factors on liver fat content.

In this respect, dietary niacin intake (nicotinic acid, vitamin B3, or its corresponding amid nicotinamide) could be a potential modulator of hepatic steatosis. Niacin inhibits lipolysis in adipose tissue, decreases the activity of diacylglycerol acyltransferase-2 (DGAT2) in hepatocytes and possibly increases hepatic lipid oxidation^11,12^. Therefore, we investigated whether dietary niacin intake predicted the change in liver fat content during a lifestyle intervention in subjects who are at increased risk for type 2 diabetes.

## Methods

### Subjects

The data analyzed in this report derive from the Tübingen Lifestyle Intervention Program (TULIP). This is a non-randomized lifestyle intervention study that was designed in 2002 and which has been performed between 2003 and 2009^13–15^. The study has been registered with the German Research Foundation as ‘KFO114’ (http://gepris.dfg.de/gepris/projekt/5396893?language=en). The study recruited individuals from the Tübingen region in Southern Germany who fulfilled at least one of the following criteria: a family history of type 2 diabetes, a BMI > 27 kg/m^2^, a previous diagnosis of impaired glucose tolerance and/or of gestational diabetes. They were considered healthy according to a physical examination and routine laboratory tests. The participants had no history of liver disease and did not consume more than 2 alcoholic drinks per day. A total of 202 subjects met the aforementioned requirements and complete data about the dietary intake and measurements of body fat distribution and liver fat content using magnetic resonance techniques at baseline and at follow-up were available. Informed written consent was obtained from all participants. The medical ethics committee of the University of Tübingen had approved the protocol which was conform to the Declaration of Helsinki.

### Lifestyle intervention

After the baseline measurements, individuals underwent 30 minutes one-to-one dietary counselling with trained dietitians. The whole lifestyle intervention had a duration of two years divided into two phases. The first phase lasted for 6 months and comprised monthly counselling visits. The second phase lasted for 18 months with a total of 6 counselling visits (one every 3 months in average). During the duration of the next 9 months, every participant had up to ten individual counselling sessions. At each visit, the participants presented a 3-day food diary and discussed the results with the dieticians. Applying established lifestyle intervention strategies^16^ counselling was aimed to reduce body weight by ≥5%, to reduce the intake of calories from fat to <30% and particularly the intake of saturated fat to ≤10% of energy consumed and to increase the intake of fibres to at least 15 g/1000 kcal. Participants were advised to avoid consuming alcohol within the frame of recommendations aiming at reducing body weight and to increase fruit and vegetables consumption within the frame of recommendations aiming at increasing fiber intake. No dietary advice about salt and coffee intake was not given. Other specific dietary recommendations were not provided. Diet composition was estimated with a validated computer program using 2 representative days of a 3-day diary (DGE-PC 3.0, Deutsche Gesellschaft für Ernährung, Bonn, Germany). Niacin intake was assessed by the computer program based upon the niacin content of each food component, and provided in mg/d. Mean daily niacin intake from diet protocols before and during the lifestyle intervention were used as variables in our models. Individuals were asked to perform at least 3 hours of moderate sports per week. Aerobic endurance exercise (e.g. walking, swimming) with an only moderate increase in the heart rate was encouraged. All subjects completed a standardized self-administered and validated questionnaire to measure physical activity and a habitual physical activity score was calculated^17^. Participants were seen by the staff on a regular basis to ensure that these recommendations were accomplished.

### Total body fat mass, body fat distribution and liver fat content

Measurements of total body-, subcutaneous- and visceral fat mass were performed by an axial T1-weighted fast spin echo technique with a 1.5 T whole-body imager (Magnetom Sonata, Siemens Medical Solutions). Liver fat content was measured by localized proton magnetic resonance (^1^HMR) spectroscopy^18^. NAFLD was defined as liver fat content >5.56%^19^.

### Peak aerobic capacity (VO_2peak_)

The individuals underwent a continuous, incremental exercise test to volitional exhaustion using a cycle ergometer. The cycle ergometer test was performed on an electromagnetically braked cycle ergometer (Ergometrics 800 S; Ergoline, Bitz, Germany). Oxygen consumption was measured using a spiroergometer (MedGraphics System Breese Ex 3.02 A; MedGraphics). VO_2peak_ is expressed as VO_2_ (ml/min) per kg lean body mass (ml∙min^−1^∙kg^−1^).

### Oral glucose tolerance test and laboratory measurements

All individuals underwent a 75 g oral glucose tolerance test (OGTT) and venous blood samples were obtained at 0, 30, 60, 90 and 120 minutes for determination of plasma glucose and insulin. Blood glucose was determined using a bedside glucose analyzer (glucose-oxidase method; YSI, Yellow Springs Instruments, Yellow Springs, CO). For measurements of insulin, blood was placed on ice after drawing, immediately transferred to the lab and subsequently analyzed. Plasma triglycerides, total cholesterol, and HDL and LDL cholesterol were measured using the ADVIA 1650 clinical chemical analyzer, and insulin was analyzed using the ADVIA Centaur immunoassay system according to the instructions of the manufacturers. Both analyzers were from Siemens Medical Solutions (Fernwald, Germany). We measured the serum free fatty acid (FFA) concentration with an enzymatic method (WAKO Chemicals, Neuss, Germany). Insulin sensitivity was calculated from glucose and insulin values during the OGTT, as proposed by Matsuda and deFronzo^20^. The adipose tissue insulin resistance index was determined as previously described^21^. The single nucleotide polymorphism (SNP) rs1944438 C > T in *DGAT2* was genotyped by direct sequencing and using the TaqMan® assay (Applied Biosystems) as previously described^8^.

### Statistical analyses

Unless otherwise stated, data are given as mean ± SE (standard error). Differences between baseline and follow-up data were tested using the Wilcoxon signed-rank test. Change of liver fat content was assessed as fold change (liver fat_post-intervention_/liver fat_pre-intervention_). Relationships were tested with forward stepwise regression (using the Bayesian Information Criterion to decide on the inclusion of a variable) and ordinary multivariable regression models. For the inclusion in the regression models, parameters with skewed distributions were logarithmically transformed to approximate a normal distribution. Effect sizes were provided as standardized beta (β) or as non-standardized estimates. The relationship of the SNP rs1944438 C > T in *DGAT2* with the change in liver fat content was studied using a dominant model. Logistic regression was applied to determine the odds ratio for achieving a resolution of NAFLD. A two-sided p-value ≤ 0.05 was considered statistically significant. The statistical software package JMP 10 (SAS Institute Inc, Cary, NC, USA) was used.

## Results

The lifestyle intervention resulted in a mean weight reduction of 2.6 kg (p < 0.0001). Total body-, subcutaneous- and visceral fat mass and glycemia also decreased, while insulin sensitivity, the HPA score, representing an estimate of the average daily physical activity, and VO_2peak_ increased. Total energy intake and the intake of calories from fat decreased. On the other hand, the intake of fibers and of calories from carbohydrates, and particularly from protein, increased. The intake of niacin also tended to increase, however, the change of niacin intake was relatively small. The largest effect of the lifestyle intervention was found for liver fat content (−32%, Table 1). Nevertheless, a large intra-individual variability was observed for this change.

**Table 1:** Subject characteristics before and after 9 months of the lifestyle intervention

Parameters	Before intervention	After intervention	Change (%)	p
Gender (Males/Females)	82/120			
Age (years)	45 (38;54)			
Duration of intervention (days)	253 (219;289)			
BMI (kg∙m^−2^)	29.0 (26.0;32.1)	27.9 (25.3;31.7)	−4%	<0.0001
Waist circumference (cm)	97.0 (87.8;104.0)	92.3 (84.0;102.1)	−5%	<0.0001
Total body fat mass _MRT_ (kg)	24.9 (17.9;32.2)	22.1 (16.1;29.2)	−12%	<0.0001
Visceral fat mass _MRT_ (kg)	2.6 (1.5;4.1)	2.0 (1.1;3.6)	−21%	<0.0001
Subc. fat mass _MRT_ (kg)	21.9 (15.4;28.8)	19.9 (13.8;26.5)	−9%	<0.0001
Liver fat content _MRS_ (%)	3.0 (1.4;6.9)	2.0 (1.0;5.0)	−32%	<0.0001
Fasting glucose (mM)	5.2 (4.8;5.6)	5.1 (4.8;5.5)	−2%	0.003
2 h glucose (mM)	6.6 (5.8;8.0)	6.4 (5.6;7.7)	−4%	0.03
Insulin sensitivity (AU)	11.8 (7.1;17.4)	13.3 (8.9;19.3)	13%	<0.0001
Total cholesterol (mg/dl)	190.5 (164.8;215.0)	186.0 (163.0;208.5)	−2%	0.04
LDL cholesterol (mg/dl)	119.0 (98.0;139.0)	115.0 (95.0;137.0)	−3%	0.003
HDL cholesterol (mg/dl)	50.0 (43.0;60.0)	52.0 (43.0;60.5)	4%	0.58
Triglycerides (mg/dl)	99.0 (76.0;137.5)	94.0 (69.0;131.5)	−5%	0.02
Fasting free fatty acids (µmol/l)	654.0 (503.0;800.0)	602.5 (467.8;726.3)	−8%	0.0002
2 h free fatty acids (µmol/l)	61.0 (43.0;93.0)	52.5 (34.0;76.0)	−14%	<0.0001
Adipo-IR_i_ (µmol/l∙pM)	31819 (20955;54918)	24608 (16020;41159)	−23%	<0.0001
Physical activity (arb. u)	8.25 (7.50;9.00)	8.50 (8.00;9.25)	3%	<0.0001
VO_2peak_ (ml∙min^−1^∙kg^−1^)	24.5 (20.2;28.5)	25.3 (21.0;30.6)	3%	0.0002
Total energy intake (kCal/day)	1925 (1628;2310)	1765 (1563;2082)	−8%	<0.0001
Calories from carb. (%)	47.2 (42.89;52.02)	48.77 (44.81;51.77)	3%	0.046
Calories from fat (%)	32.48(28.22;37.11)	30.96 (27.90;34.67)	−5%	0.002
Calories from protein (%)	15.38 (13.80;17.48)	16.15 (14.86;17.94)	5%	0.0001
Fibers (g/d)	22.25 (17.88;26.82)	25.14 (21.47;30.15)	13%	<0.0001
Niacin intake (mg/d)	12.3 (9.8;15.9)	13.1 (10.7;16.2)	6%	0.06

Data are given as medians (interquartile range). P values were calculated with the Wilcoxon signed rank test. Adipo-IR_i_, adipose tissue insulin resistance index; BMI, body mass index; MRT, magnetic resonance tomography; MRS, magnetic resonance (MR) spectroscopy; Vo_2peak_, peak oxygen uptake.

In the study, 32% of male subjects and 31% of female subjects had baseline niacin intakes fulfilling dietary reference intakes (16 g/d and 14 g/d, respectively^22^). Niacin intake correlated positively with total energy intake, the proportion of proteins, and negatively with the proportion of carbohydrates (see Supplementary Table 1).

Linear regression models demonstrated that a higher daily niacin consumption at baseline associated with a larger decrease of liver fat content (β = −0.19, p = 0.004) adjusted for liver fat content at baseline. The association of niacin intake with change of liver fat content remained statistically significant (β = −0.21, p = 0.008) also after additional adjustment for sex, age, total body fat mass at baseline and at follow-up and total daily energy intake at baseline (Table 2, model 1). Niacin intake did not change significantly during lifestyle intervention (Table 1). Also, niacin intake at the end of lifestyle intervention did not significantly associate with liver fat reduction in the above model (p = 0.26).

**Table 2:** Determinants of the change in liver fat content in multivariable regression models.

	Estimate	SE	p value
**Model 1**			
Gender (female)	−0.09	0.05	0.08
Age	0.08	0.16	0.62
Liver fat content_baseline_	−0.27	0.04	<0.0001
Total fat mass_baseline_	0.02	0.17	0.90
Total fat mass_follow-up_	0.25	0.12	0.03
Total energy intake	−0.02	0.22	0.93
Niacin intake	−0.38	0.14	0.008
**Model 2**			
Gender (female)	−0.09	0.05	0.08
Age	0.15	0.16	0.36
Liver fat content_baseline_	−0.29	0.04	<0.0001
Total fat mass_baseline_	0.02	0.17	0.91
Total fat mass_follow-up_	0.24	0.12	0.04
Total energy intake	0.10	0.22	0.67
Niacin intake	−0.58	0.15	0.0002
rs1944438 C > T	−0.14	0.04	0.003
Niacin intake*rs1944438 C > T	−0.26	0.12	0.035

Next, we determined whether the change of liver fat content depended on the intake of other nutrients or the amount of physical activity. For this, we ran a forward stepwise regression analysis investigating the impact of total energy intake, intake of calories from carbohydrates, fat and protein, intake of fibers, niacin and physical activity at baseline, to predict the change of liver fat content. In this model, the intake of niacin strongly predicted the change of liver fat content and only the intake of calories from carbohydrates, was entered as another considerably weaker predictor into the stepwise regression model (Table 3). In addition, in a multivariable model, the change of liver fat content, after adjustment for total daily energy intake, intake of calories from carbohydrates and physical activity, all at baseline and at follow-up, the niacin intake at baseline was predictive of the change of liver fat content (Table 4).

**Table 3:** Baseline nutrient intake and physical activity as putative predictors of the change of liver fat content in a forward stepwise multivariate regression model.

	Estimate*	F-Ratio	p value
Liver fat content_baseline_**	−0.22	31.6	<0.0001
Niacin intake**	−0.33	8.5	0.004
Total energy intake (kCal)**		0.06	0.81
Carbohydrate intake (g/day)		5.3	0.02
Fat intake (g/day)		3.6	0.06
Protein intake (g/day)		2.24	0.14
Fiber intake (g/day)		0.24	0.62
Physical activity		0.07	0.79

^*^Only estimates of variables in the final model are shown.

^**^Natural-log transformed variables.

**Table 4:** Independent effect of niacin intake from other nutrients intake and from physical activity on the change of liver fat content in a multivariable regression model.

	Estimate	SE	p value
Liver fat content_baseline_	−0.33	0.04	<0.0001
Niacin intake_baseline_	−0.44	0.15	0.003
Total energy intake_baseline_	−0.06	0.24	0.79
Total energy intake_follow-up_	0.13	0.26	0.62
Carbohydrate intake_baseline_	−0.01	0.007	0.04
Carbohydrate intake_follow-up_	−0.0004	0.008	0.96
Physical activity_baseline_	0.02	0.05	0.67
Physical activity_follow-up_	−0.06	0.05	0.29

Finally, using our initial model, we tested whether niacin intake predicted the change of liver fat content also after adjustment for the established predictor of liver fat content, aerobic fitness^10^. Niacin intake at baseline remained significantly associated with the change of liver fat content, adjusted for baseline liver fat content, sex, age, total body fat mass at baseline and at follow-up, and total daily energy intake and VO_2peak_ at baseline (n = 190, β = −0.20, p = 0.02).

### Niacin intake

**Figure 1:**
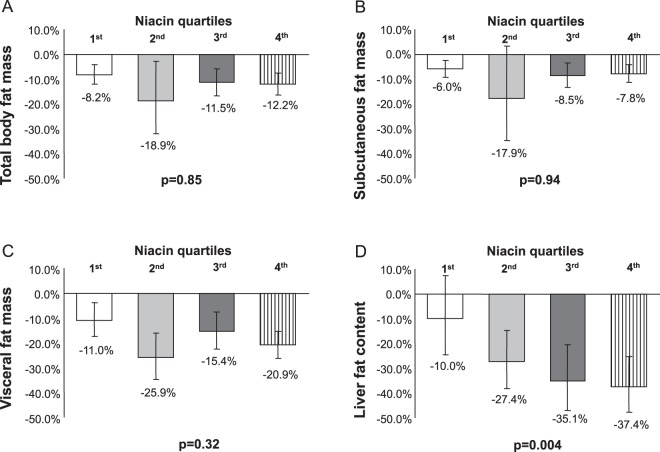
Relationships between baseline niacin intake and changes in fat compartments. Change in total body-, subcutaneous- and visceral fat mass (**A–C**) and liver fat content (**D**) during the lifestyle intervention stratified across quartiles of niacin intake. Quartile 1 represents the stratum with the lowest niacin intake (median, 7.7 mg/d), and quartile 4 represents the stratum with the highest niacin intake (median, 19.5 mg/d). Bars indicate geometric means, error indicators show 95% confidence intervals for the geometric means. P-values are given as p for trend.

To better depict the relationships of niacin intake with changes of total body fat mass, body fat distribution and liver fat content, we divided the subjects into quartiles according to niacin intake at baseline. A significant association of niacin intake quartiles was observed with the change of liver fat content, while total body-, subcutaneous- and visceral fat mass did not associate with the niacin intake (Fig. 1).

**Figure 2:**
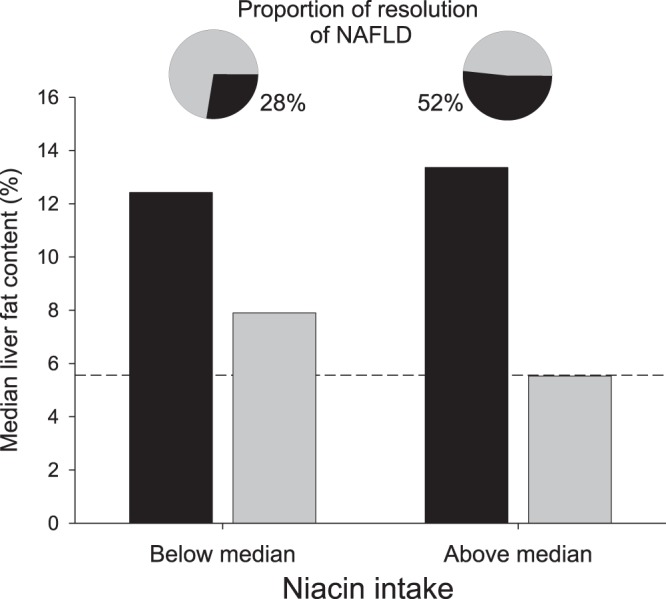
Relationships between baseline niacin intake and resolution of NAFLD. In subjects with non-alcoholic fatty liver disease (NAFLD) before lifestyle intervention, liver fat content before (black bars) and after (grey bars) lifestyle intervention is depicted across groups with lower (below median) and higher (above median) niacin intake. The dashed line represents the percent liver fat content above that NAFLD is present (5.56%). In more participants NAFLD resolved during the lifestyle intervention in the higher niacin intake group than in the lower niacin intake group (p = 0.03, pie charts with grey slices representing the proportion of subjects experiencing a resolution of NAFLD.

From 202 subjects studied, 58 persons had NAFLD at baseline, with a median liver fat content of 12.6% (IQR 7.9–17.6). NAFLD resolved in 23 subjects during the lifestyle intervention. For one standard deviation increase in niacin intake, the odds ratio for resolution of NAFLD was 1.77 (95% CI, 1.00–3.43). To depict these relationships in the 58 subjects with NAFLD, they were then stratified by the median niacin intake. While liver fat content at baseline was similar in both groups, more subjects experienced a resolution of NAFLD in the group having a higher than in the group having a lower niacin intake (Fig. 2). In the subset of participants with NAFLD at baseline, there was a significant inverse association of niacin intake with fold-change liver fat content (using the same model covariates as given in Table 2, model 1, b = −0.76 + −0.32, p = 0.02 for log-transformed baseline niacin intake). Of note, the absolute effect size of niacin intake increased in the subgroup with fatty liver, indicating a higher reduction of liver fat content for a given amount of niacin intake in these subjects, compared to the whole dataset.

We observed an interaction of baseline niacin intake with BMI change during lifestyle intervention on the reduction of liver fat (p = 0.04, see Supplementary Fig. 1A,B), suggesting that the baseline-niacin dependent reduction of liver fat content was higher in participants loosing more weight.

Because we previously found that carriers of the minor T allele of the SNP rs1944438 in *DGAT2* had less decrease in liver fat content during our lifestyle intervention compared to homozygous carriers of the major C allele^8^, and niacin is known to inhibit DGAT2 activity in hepatocytes^12^, we investigated whether this SNP interacts with niacin intake on the change of liver fat content. In fact, the interaction term niacin*SNP was significant (p = 0.035) (Table 2, model 2) and niacin more strongly predicted a decrease of liver fat content in homozygous carriers of the major C allele (n = 68; β = −0.90, p = 0.0008) than in carriers of the T allele (n = 125; β = −0.22, p = 0.24).

As niacin intake may also influence insulin sensitivity, we also tested whether niacin intake has an independent effect on the change of insulin sensitivity during the lifestyle intervention. Niacin intake at baseline was not associated with the change of insulin sensitivity after adjustment for baseline insulin sensitivity, sex, age, energy intake and total body fat mass at baseline and at follow-up (β = 0.14, p = 0.13). Also, no relationship of niacin intake with the change of the adipose tissue insulin resistance index, adjusted for the baseline adipose tissue insulin resistance index, sex, age, energy intake and total body fat mass at baseline and at follow-up was observed (β = −0.002, p = 0.99).

## Discussion

Our data indicate that higher dietary niacin intake predicts a larger decrease of liver fat content during a lifestyle intervention. These data suggest that an elevated dietary niacin intake may facilitate the effect of lifestyle intervention on the treatment of hepatic steatosis in humans.

The initial hypothesis about a relationship of niacin intake with liver fat content was based on data about the intricate role of niacin in lipid metabolism. While niacin supplementation increases HDL-cholesterol, it reduces triglyceride levels, also lowering LDL-cholesterol and lipoprotein(a). The full mechanism of niacin action still remains elusive. Based upon available data, the reduction of hepatic triglyceride content that was seen in our subjects with high niacin intake could potentially be attributed to three possible mechanisms. First, niacin inhibits lipolysis in peripheral adipose tissue through its action on the specific nicotinic acid receptor hydroxy-carboxylic acid (HCA) receptor 2 (HCA2)^12^, thereby, reducing the flux of free fatty acids to the liver. However, the niacin-mediated suppression of lipolysis is subject to a rebound phenomenon^23^. This consecutive elevation of plasma FFAs most probably cannot be abrogated by the use of an extended-release niacin formulation^23^. Second, *in vitro* niacin directly and non-competitively inhibits DGAT2, the hepatic DGAT isoenzyme responsible for the committing step of triglyceride synthesis^24^. Third, niacin intake is associated with changes in the microbiome which plays an important role in human metabolism^25^.

The effect of niacin on liver fat content has so far been examined by two randomized controlled trials using extended release niacin preparations. In the first study comprising 9 participants on niacin and 9 on placebo, no niacin-associated change of liver fat content was observed^23^. In the second study involving 39 participants, niacin was shown to reduce liver fat content by a mean of 47.2% during the study duration of 23 weeks^24^. However, a reduction of insulin sensitivity was observed in both studies.

In our study, high niacin intake during lifestyle intervention was associated with a larger reduction of liver fat content without affecting insulin sensitivity.

What may explain the different findings between these studies in respect to insulin sensitivity? The studies by Fabbrini *et al*.^23^ and Hu *et al*.^24^ used pharmacologic niacin doses titrated to 2000 mg/day and 2000 mg/week, respectively. In contrast, dietary niacin intake amounted to up to 2 orders of magnitude less in our study (median, 12.3 mg/day, IQR 9.8; 15.9). Because a rebound elevation of FFAs is thought to be among the possible causes of niacin-induced decrease in insulin sensitivity when using higher doses^26^, we speculate that these effects were absent in our study. In support of this hypothesis, niacin intake was not associated with the change of adipose tissue insulin resistance index, which is an estimate of adipose tissue lipolysis. Thus, it can be speculated that the uptake of niacin by nutrients in lower doses less likely result in an FFA rebound. In context with recent data from Fangmann *et al*., we could also hypothesize that diet-bound niacin is predominantly released in specific intestinal segments such as the terminal ileum^25^. Niacin effect in the distal gut could directly or via the microbiome indirectly be responsible for reduction of liver fat.

Furthermore, we pinpointed a gene-nutrient interaction. A higher niacin intake was predictive of a larger decrease in liver fat content predominantly in homozygous carriers of the major C allele of rs1944438 in *DGAT2*. Interestingly, Hu *et al*. found that the effect of niacin treatment on the change of liver fat content also depended on a SNP in *DGAT2*^24^. These data mechanistically support *in vitro* results on a direct effect of niacin to inhibit DGAT2 activity in hepatocytes. In addition, the fact that niacin intake predicted the decrease of liver fat content independently of the SNP rs1944438 in *DGAT2* indicates that niacin may regulate liver fat content also by other mechanisms.

Our study has limitations. This work is retrospective analysis of a cohort study performed several years ago. Thus, dietary recommendations that may derive from the time when the study was conducted (2003–2009) may not necessarily be applicable to the setting today. Also, subjects were not asked to increase their niacin intake in a randomized controlled setting and, therefore, could only perform a retrospective analysis. However, as many longer lasting nutritional intervention strategies are prone to false reporting and poor compliance of the participants, we did not introduce such a bias that may largely affect the results. The results should be interpreted in the context of the cohort’s elevated type 2 diabetes and cardiovascular risk. We also did not account for intestinal niacin production in our work. It has been long known that intestinal bacteria can synthesize niacin^27–29^. According to a recent estimate, 27% of the dietary reference intake of niacin is provided by human gut bacteria^30^ Despite careful adjustment of our models for possible confounders, we cannot exclude that a high dietary niacin intake may reflect a yet unknown beneficial behavioral or nutritional aspect that resulted in a larger decrease in liver fat content in these subjects. The fact that the niacin intake was associated with the change of liver fat content independently of the changes of body fat content, energy intake and physical activity, makes this hypothesis less probable. The interaction of niacin intake with rs1944438 in *DGAT2* affected liver fat content. Since DGAT2 is the probable mediator of niacin’s effects on triglyceride synthesis^24^, this strengthens the notion that the observed association was a result of differences in niacin intake. While DGAT2 constitutes the most probable link between niacin and hepatic steatosis via regulation of hepatic triglyceride synthesis, other potential mechanisms include the expression of ATP synthase beta chain in the cell surface of hepatocytes and the transcription of ATP-binding cassette transporter A1 (ABCA1) which promotes apoAI lipidation^31,32^. However, we have both precise measurements of liver fat content and well documented data about food diaries, enabling analyses in the present setting. Furthermore, the fact that the relationship of dietary niacin intake with the change of liver fat content was not only present in all 202 subjects, but also in the subgroup of 58 subjects with NAFLD at baseline, supports that the chance of the results being subject to a type I error is relatively small.

In conclusion, our study demonstrates that higher niacin intake favorably modulates the effect of a lifestyle intervention on liver fat content. This suggests a beneficial effect of higher niacin consumption in reaching the therapeutic goals of a lifestyle intervention to improve hepatic steatosis. Intervention studies testing the influence of niacin-fortified foods on outcomes of a lifestyle intervention to treat NAFLD are now needed to validate this concept.

## Supplementary information

Supplementary Material

## Data Availability

The dataset analysed during the current study is not publicly available due to local data protection rules, but it is available from the corresponding author on reasonable request.
