# Supplementary material for: Dietary Niacin Intake Predicts the Decrease of Liver Fat Content During a Lifestyle Intervention
Source: Sci Rep. 2019 Feb 4;9:1303. doi: 10.1038/s41598-018-38002-7 (PMC6362104; doi:10.1038/s41598-018-38002-7)
Supplement: Supplementary file 1 — Supplementary Material [file 41598_2018_38002_MOESM1_ESM.docx]

# Supplementary Material of

Dietary niacin intake predicts the decrease of liver fat content during
a lifestyle intervention

Katarzyna Linder, MD^1,2,3^_,_ Caroline Willmann, MD^1,2^, Konstantinos Kantartzis, MD^1,2,3^, Jürgen Machann, PhD^2,3,4^, Fritz Schick, MD, PhD^2,3,4^, Marjo Graf^1^, Sabine Kümmerle^1^, Hans-Ulrich Häring, MD ^1,2,3^, Andreas Fritsche, MD ^1,2,3^, Norbert Stefan, MD ^1,2,3^, Róbert Wagner, MD^1,2,3^

## Supplementary Figure 1 A and B

Tertiles of change of BMI during lifestyle intervention (A) with corresponding linear regression models representing the interactions between BMI change and baseline niacin intake (B) on fold change of liver fat (interaction p=0.04).


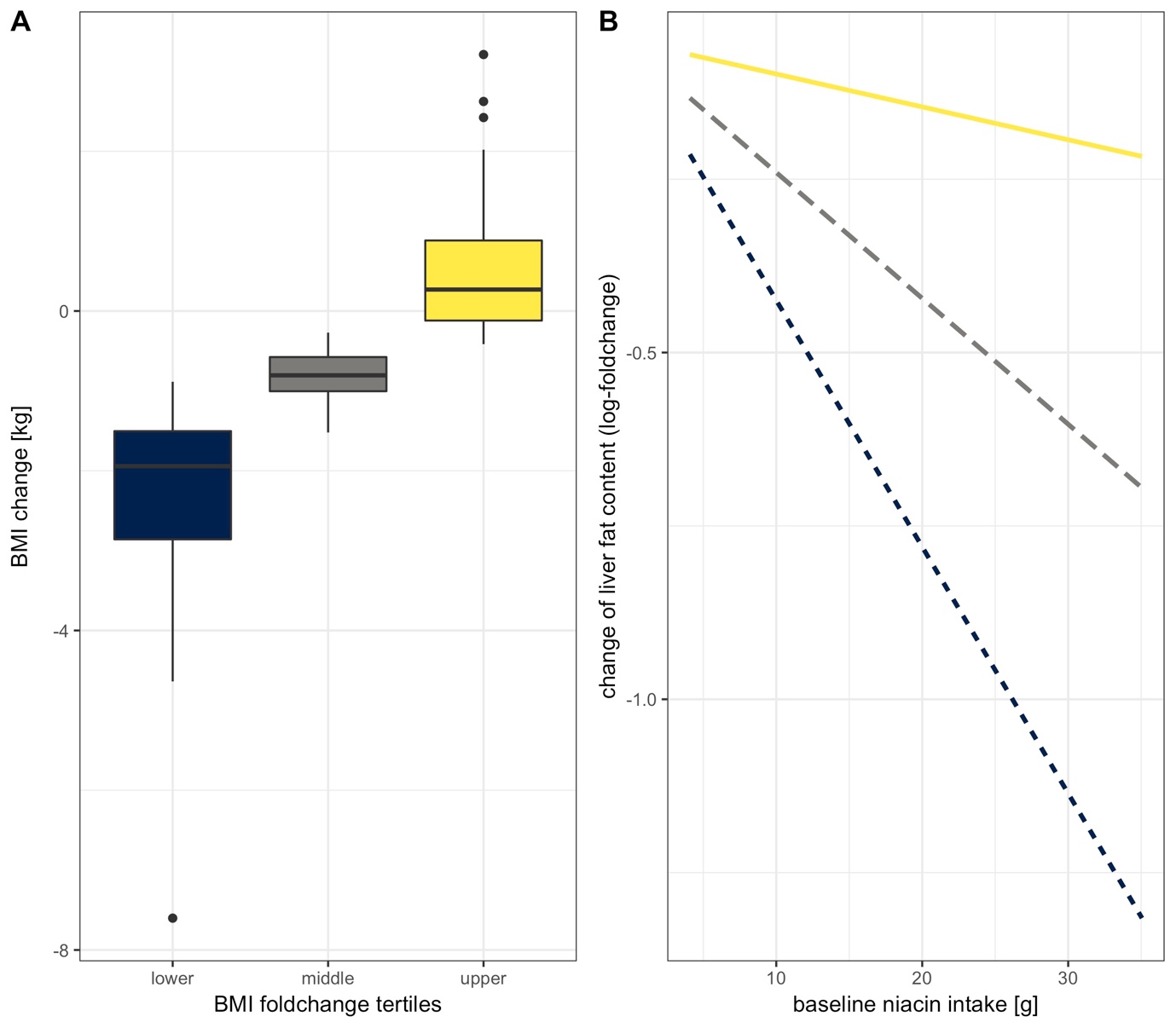


## Supplementary Table 1

Correlation of niacin intake (mg/day) with energy intake and macronutrient composition of the diet at baseline (before lifestyle intervention) in the TULIP study (N=202)

|  | Correlation coefficient (r) | p-value |
| --- | --- | --- |
| Energy intake (kCal/day) | 0.55 | <0.0001 |
| Carbohydrates (% of energy intake) | -0.33 | <0.0001 |
| Fats (% of energy intake) | 0.08 | 0.28 |
| Proteins (% of energy intake) | 0.31 | <0.0001 |
